# Performance Assessment Indicators for Comparing Recreational Services of Urban Parks

**DOI:** 10.3390/ijerph18073337

**Published:** 2021-03-24

**Authors:** Yang Yang, Zhifang Wang, Guangsi Lin

**Affiliations:** 1 Department of Landscape Architecture, School of Architecture, South China University of Technology, Guangzhou 510641, China; yangyyla@outlook.com; 2 College of Architecture and Landscape Architecture, Peking University, Beijing 100871, China; zhifangw@pku.edu.cn; 3 State Key Laboratory of Subtropical Building Science, South China University of Technology, Guangzhou 510641, China; 4 Guangzhou Municipal Key Laboratory of Landscape Architecture, South China University of Technology, Guangzhou 510641, China

**Keywords:** urban park management, cultural ecosystem services, evaluation indicator, high-performance landscape, park use, recreational physical activity

## Abstract

Parks can offer varied services to human well-being, including recreational services (RS); however, there is insufficient understanding of park differences concerning the actual performance of their varied RS. To fill this gap, this study aimed to develop a set of performance indicators as a tool for comparing the functional efficacy of RS among different parks. The indicators covered three aspects of RS: recreational usage of various physical activities, their recreational satisfaction level and the collective performance rating. These indicators were applied in a case study of four parks in Guangzhou, China, based upon on-site observation and a questionnaire survey. The functional difference of these indicators was compared and the collective indicator was found to be able to effectively capture different efficacies of urban parks in supporting varied RS. Results show all the parks were far from reaching the maximum performance of designed RS, so it is worthy of consideration by urban managers to further improve their RS efficacy. In addition, the overall spatial design and configuration were inferred to be essential for improving the RS efficacy of urban parks, but not park size nor ornamental vegetation. The findings offered valuable evidence for the optimal spatial design and management of urban parks.

## 1. Introduction

Urban parks can supply a wide range of ecosystem services [1], including recreational services (RS), which are essential among cultural ecosystem services [2,3,4]. RS refers to a wealth of opportunities for outdoor leisure activities that urban green spaces offer to urban residents [5]. Abundant evidence has shown that park-based physical activities provide people substantial mental and physical health benefits, like stress reduction, attentional improvement and chronic disease prevention [6,7,8,9], which are paramount to public health, so urban parks play a vital role in creating the eco-liveable city [10,11]. Generally, in urban planning and management, the number, area and spatial distribution of parks are common indicators to evaluate the service levels of urban parks [12,13,14]. For example, in China, parkland area per capita, parkland service radius coverage and comprehensive park index per 10,000 people are important assessment indicators for National Garden City [15]. Nevertheless, more and more studies have pointed out that park quality has a more important association with residents’ health than park quantity [16,17,18,19,20]. High-quality parks with good landscape settings and recreational functions can encourage a variety of users to walk, play and socialise [18,21,22]; however, a few built parks, even well-sited and good-sized ones, were underutilised [23,24]. Therefore, more than presence, the high-quality internal spaces of a park that can boost a range of recreational physical activities will produce higher efficacy of RS, contributing to urban public health [18,25].

Performance assessment is a common research approach to test the effectiveness with which design solutions fulfil their purpose and provide a scientific basis for future design and management decision-making [26]. At the layer of regional planning, the indicators of accessibility [27], balance of supply and demand [12] and service radius [28] are widely used to measure the RS level of park provision, providing evidence for the park’s spatial layout optimisation. In addition to the external accessibility of the park from residences [29,30], the internal spatial configurations of a park greatly affect its overall recreational use [31,32]. Hence, performance indicators on the RS function of urban parks are urgently needed to assess how well the overall designed recreational spaces of a park achieve its RS goals. The assessment results can provide evidence to park designers and managers for refined management and (re)design of parklands [33], especially in high-density urban areas where land resources are under stress. In the study of built environment performance evaluation, space occupancy and user perception are important variables to measure the service quality of built public spaces, including urban parks, since “use” and “used well by people” are conditions for a successful public space project [34]. Research to date has mainly focused on revealing the spatial attributes associated with user needs or preferences [35,36,37,38] by measuring the visitor numbers and user satisfaction of a park [26,39,40,41]. Many studies have reported spatial factors such as area size, maintenance, facilities, safety, atmosphere and so on for encouraging physical activities [21,42,43,44,45] to inform park design and management. However, there is a lack of exploration on how the overall spatial attributes of a park affect its RS efficacy. As a spatial product serving public life, park construction tends to take into account the diverse RS needs in a limited land area. Only one study that we know of focused on the associations of spatial attributes with certain user groups from aspects of demographics or cultural contexts [38]. It is still unclear if there are significant differences concerning the actual performance of varied RS of parks with different spatial attributes. To fill this gap, the performance indicators for comparing the RS of different parks from an overall perspective are considered crucial.

The typical evaluation methods of visitor statistics or user satisfaction in available studies failed to accurately reflect the overall RS performance for comparison of different parks, because the performance of RS involves the collection of participants and their satisfaction. The purpose of urban parks, especially large ones, is to serve different kinds of activities and high service quality exists when recreation opportunities meet the needs of various visitors [46]. However, it was reported that Chinese young people were less likely to use parks [47] and this was related to a lack of attractiveness for young adults of urban parks in China [48]. As a result, the diversity of activity types that visitors engaged in should also be an important index to assess a park’s service quality. On the other hand, in terms of satisfaction, different activity groups have different criteria for recreational satisfaction due to their different spatial demands and preferences. Studies simply surveying users’ satisfaction scores without considering the differences between activity categories have failed to truly reflect the actual performance of park spaces satisfying all kinds of recreational users’ needs. In addition, some studies found the park size had a strong association with the number of users [49,50]. Obviously, it is not difficult to understand that a larger park with more vegetation or recreational grounds can attract and contain more users, but regarding efficacy, it is unclear if a larger park would perform better than a smaller one. Thus, more collective indicators covering varied RS based on a unified area are more valid for park comparison.

This study aimed to develop comparative performance assessment indicators by analysing the RS function mechanism, involving the RS function for various recreational physical activities and service efficacy of a park. Then, these indicators were applied in a case study of four urban parks with different overall spatial attributes in central Guangzhou, Guangdong Province, China, to reveal their performance differences in RS and explore the underlying influencing factors. The study is expected to provide an effective performance assessment method to inspect the overall RS efficacy of urban parks and to provide scientific evidence supporting the optimal spatial design and management of parks.

## 2. Materials and Methods

### 2.1. Performance Assessment Indicators for Recreational Services of Urban Parks

**Figure 1:**
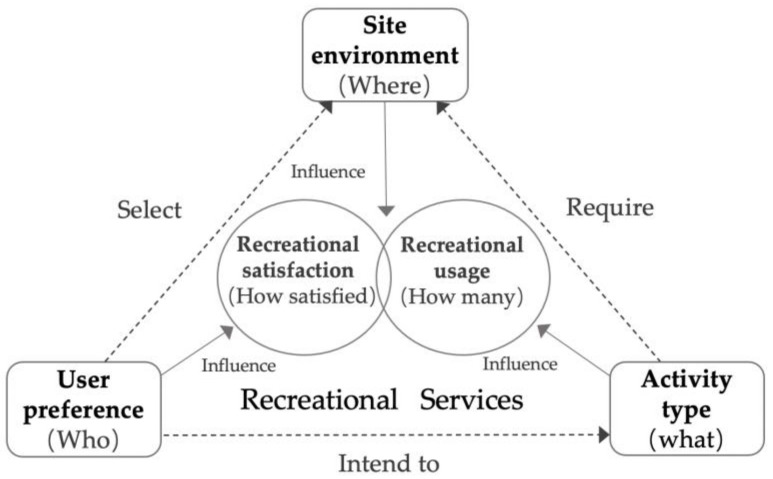
The recreational services function mechanism in a park.

The RS of park spaces is mainly embodied in recreational usage and satisfaction [51,52]. Conceptually, the recreational usage involves the number of recreational users in the park spaces and the recreational satisfaction relates to the individual perception about to what degree the user is satisfied within the park settings for certain physical activity. Theoretically, to establish fine measurement indicators linking space, activity and performance, we decomposed the RS function mechanism in a park, as shown in Figure 1 and suggested the relationships among site environment, activity type and user preference, which are the main influencing factors on the recreational usage and satisfaction.

Specifically, (1) site environment, the “where” of space in a park, is usually selected by “who” according to user preference and required by “what”, a certain activity the user intended to conduct. (2) User preference can influence the recreational satisfaction, that is “how satisfied” the users are with the recreation experience. (3) Various activities have different venue requirements, which indirectly influence the site usage and “how many” recreational users, reflecting the occupancy of the site environment in a park. Taken together, (4) the recreational usage and satisfaction constitute the functional performance of RS of the site environment.

**Figure 2:**
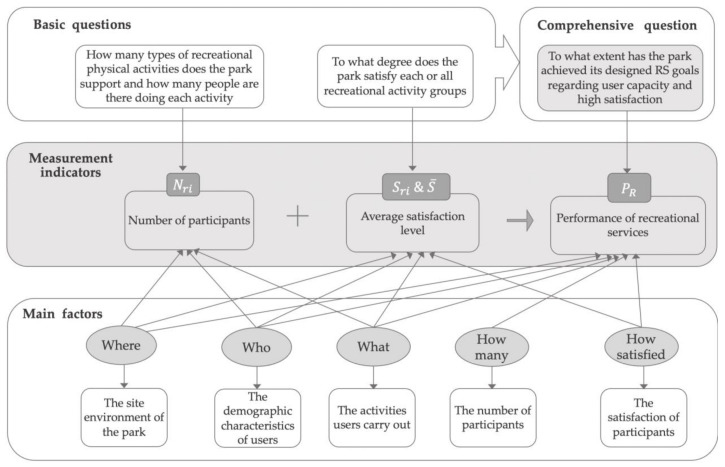
The conceptual framework of performance measurement indicators on recreational services.

Based on the above main factors in the function mechanism of RS, the measurement indicators are formulated by raising unified research questions. They are two basic questions: “How many types of recreational physical activities does the park support and how many people are there doing each activity?”; “To what degree does the park satisfy each or all recreational activity groups?” The two are combined into a collective question on RS efficacy: “To what extent has the park achieved its designed RS goals regarding user capacity and high satisfaction?” To answer these questions, the above-mentioned main factors of “who”, “what”, “where”, “how many” and “how satisfied” are combined to derive two basic indicators and one collective indicator. The conceptual framework of performance measurement indicators on RS is shown in Figure 2.

The indicators, equations and their detailed analysis are presented as follows.

(1) Number of participants: $N_{ri}$

The number of visitors participating in one type of recreational activity group per hour is represented by $N_{ri}$. The subscript *ri* represents different types of recreational activity groups, in which *i* = 1, 2, …, x, which means there are x types of recreational activity groups in a park.

(2) Average satisfaction level:$\;S_{ri}$ and $\bar{S}$.

This study collects the satisfaction scores of different recreational activity groups by the questionnaire survey method. The score is from 1 to 5 (step = 1; the full score is 5). The satisfaction level for one type of recreational activity group is represented by $S_{ri}$. The subscript *ri* has the same meaning as above. $S_{ri}$ can be calculated by the following equation. When all participants in a group are very satisfied (full score), the $S_{ri}$ value will be 1.

(1) $$S_{ri}=\frac{Average\;score\;of\;one\;type\;of\;recreational\;activity\;group\;}{5}$$

The average satisfaction level of all types of recreational activity groups in a park is represented by $\bar{S}$, which can be calculated by the following equation. When all groups are very satisfied (full score), the $\bar{S}$ value will be 1.

(2) $$\bar{S}=\frac{1}{x}\overset{x}{\underset{i=1}{\sum }}S_{ri}$$

(3) Performance of recreational services: $P_{R}$

The purpose of building a park is to attract more people to the park to carry out various recreational activities and obtain a satisfactory leisure experience. In this process, there are two important indicators: the number of participants $\sum N_{ri}$ and satisfaction level $\bar{S}$. However, the overall performance of RS in a park cannot be evaluated only by $\sum N_{ri}$ or $\bar{S}$.

For example, a park is designed to serve 1000 people. If this park attracts 1000 or even more people to carry out recreational activities, we cannot say that the performance of RS of this park is greater than 100%. This is because the satisfaction of individual visitors may not be that good. On the other hand, if the park only attracts 500 people to carry out recreational activities, even if the satisfaction of each of these 500 visitors is 100%, we cannot just say that the overall performance of RS in this park is 100%. This is because this park does not attract enough people (equal to or greater than the design value), which, to a certain extent, indicates that the park’s RS has not reached the maximum efficacy.

In summary, there must be a trade-off between $\sum N_{ri}$ and $\bar{S}$. It needs a comprehensive indicator to reflect the overall performance of the RS of a park. This paper defines the so-called comprehensive indicator as $P_{R}$, which is calculated by the following equation.

(3) $$P_{R}=(\overset{x}{\underset{i=1}{\sum }}N_{ri}\cdot S_{ri})/\left(\frac{S_{t}}{S_{a}}\right)$$

In the above equation, $S_{a}$ is the design activity area per capita of a park and $S_{t}\;$is the total recreational area in a park. In this paper, $S_{a}$ adopts a value of 30–60 m^2^, which is the minimum value of land area per capita in a comprehensive park according to the “Code for design of public parks” [53] in China. When the park has water recreational area, the water area per capita is calculated as 150–250 m^2^ [53]. In other countries or regions, the value of $S_{a}$ can be determined according to the corresponding park design specifications. $S_{t}$ is the sum of land area or water area in a park.

$P_{R}$ combines $N_{ri}$ and $S_{ri}$ into one equation. When all visitors are very satisfied ($\bar{S}$ = 1), it refers to the ratio of the number of visitors that one park can attract to the design number of visitors. More importantly, $P_{R}$ provides a quantitative indicator that can be used to compare the overall RS performance level between different parks.

### 2.2. Study Sites and the Spatial Attributes of the Case Parks

Guangzhou is the capital of Guangdong Province and a central city of the Pearl River Delta Metropolitan Area in southern China. Together with Beijing, Shanghai and Shenzhen, Guangzhou is among the four most powerful cities in mainland China. It has a permanent population of 15.3059 million, with an urbanisation rate of 86.46% by the end of 2019 [54]. At present, Guangzhou has jurisdiction over 11 municipal districts; among them, the urbanisation rate of four districts—Liwan, Yuexiu, Haizhu and Tianhe—reached 100%. By 2019, the parkland area per capita of Guangzhou was 17.96 m^2^, which is more than two of the lowest standards of National Garden City. The statistics of the amount of park construction of Guangzhou in the main years are shown in Table 1. The growth rate of park construction has slowed down since 2010 and remained unchanged from 2016 to 2019. Thus, the construction of parks in Guangzhou has entered the stage of quality development from quantity expansion.

**Figure 3:**
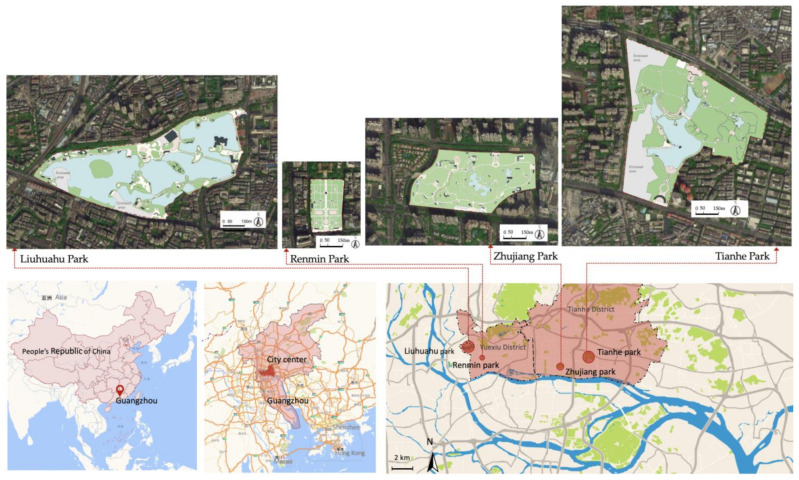
Locations and master plans of parks studied in Guangzhou, China.

In the case study, we focused on the relatively more attractive comprehensive park, which is a large park type serving various recreational needs in the classification of urban green space in China. Moreover, to minimise the possible interference of external factors such as traffic, land use and economic and social factors on the utilisation of parks, the parks close to public transport and residential areas in high-density central urban areas were targeted. Finally, the four comprehensive parks selected in this study, were Liuhuahu Park, Renmin Park, Tianhe Park and Zhujiang Park, respectively located in the Yuexiu district and Tianhe district, the core of Guangzhou, as shown in Figure 3.

**Figure A1:**
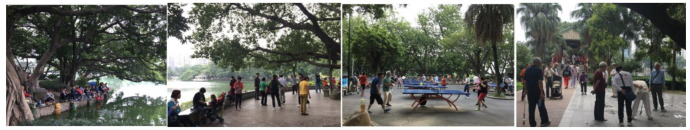
Recreation spaces in Liuhuahu Park.

The four parks varied in size and recreational spaces. Their master plans are seen in Figure 3 and some recreational spaces are shown in Appendix A. Liuhuahu Park was a large-scale comprehensive park that integrated water storage and flood control functions with recreation functions, covering an area of 54.43 hm^2^, of which the lake area accounted for 57.6%. The recreational spaces of Liuhuahu Park were rich and diverse (partly shown in Figure A1), including a leisure square, pavilion, song stage, lakeside plank, children’s playground, fitness equipment, ball game field, fishing island, theme garden and exhibition. According to the functions of recreational spaces, Liuhuahu Park had a variety of designed RS, including sightseeing, fitness, singing, playing chess, children’s playground and fishing.

**Figure A2:**
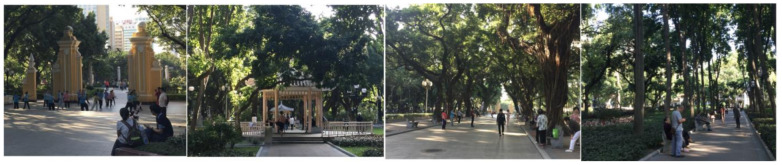
Recreation spaces in Renmin Park.

Renmin Park was the earliest comprehensive park, known as the First, Park of Guangzhou and had an area of 6.51 hm^2^ with geometric symmetry of spatial layout. The recreational spaces of Renmin Park were mainly composed of a leisure square, art square, bench and pavilion (partly shown in Figure A2). Accordingly, Renmin Park had a few designed RS, including sightseeing, dancing, singing and playing musical instruments.

**Figure A3:**
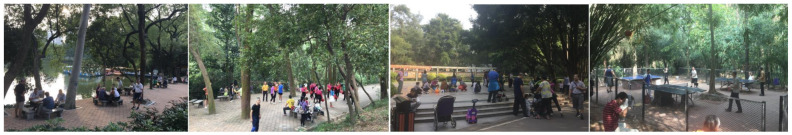
Recreation spaces in Tianhe Park.

Tianhe Park was formerly a suburban forest park. On the basis of the original mountain forest and lake, a variety of recreational spaces were set up. It had a total area of 70.7 hm^2^, of which the water area accounted for about 10 hm^2^ and the vegetation coverage rate was over 90%. Tianhe Park had different kinds of recreational spaces (partly shown in Figure A3), including a leisure square, pavilion, song stage, waterfront, children’s playground, fitness equipment and theme garden, which contributed to various designed RS of sightseeing, fitness, singing, playing chess and children’s playground.

**Figure A4:**
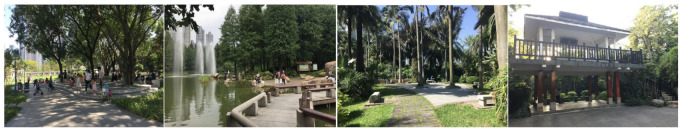
Recreation spaces in Zhujiang Park.

Zhujiang Park was built on a flat farmland and low-lying land. Its current landscape pattern was completely artificially formed by digging lakes and piles of mountains, creating an exquisite local natural garden, with a total area of 27.4 hm^2^, of which the land area was about 88.9%. The recreational spaces of Zhujiang Park were mainly composed of a leisure square, pavilion, song stage, waterfront, children’s playground, fitness equipment and theme garden (partly shown in Figure A4), which contributed to various designed RS of sightseeing, fitness, singing, playing chess and children’s playground.

### 2.3. Data Collection and Analysis

According to the indicators mentioned above, the required data include the recreational activity types, average user number and satisfaction score of each activity type and total recreational area in a park. To accurately record the type and number of recreational activities conducted in the parks, we adopted the method of field observation. A cross-sectional questionnaire survey method was used to obtain the satisfaction scores of different recreation groups based on their recreation experiences. The area of recreational sites in the park plan was mainly identified and counted based on computer technology.

#### 2.3.1. Systematic Observation

This study adopted the on-site systematic observation method via tabular record, which was designed to capture the details of users, including their demographic characteristics, activities engaged in and specific sites, which are more applicable for a large park than SOPARC (System for Observing Play and Recreation in Communities) [23]. Considering the large area of the parks and the efficiency of observation investigation, we divided the whole recreational area of each park into several space units assigned to three to five investigators. The division of space units was mainly the combination of recreational space nodes that can be connected by internal trails or other landscape elements, rather than through the main road and forms numerous independent recreational space cluster areas. It needed to be pointed out that the scopes of the recreational spaces were specially restricted by the areas where people can enter, stay, or carry out activities. However, the main traffic roads of the park were not included in the study, because it was difficult to define the user behaviour. Furthermore, the various space nodes of space units were subdivided into nine specific types based on the characteristics of common landscape elements, including landscape architecture, square or open space, water platform, landscape bridge, stone setting, flower ponds or flower paths, landscape paths, fitness trails and artificial facilities. This kind of spatial division intended to facilitate the observation work distribution and data coding in the later period of research.

Considering the time period when most recreational groups generally use urban parks, the observation survey was conducted at 8:30–9:30, 9:30–10:30, 10:30–11:30, 15:30–16:30, 16:30–17:30 and 17:30–18:30 in one day, for a total of six time periods. See Appendix B for the template of the observation record sheet. The investigators recorded the usage of each space unit and node within 2–5 min in an hour period based on on-site observations. The recorded content includes the demographic and activity characteristics of the observed crowd. The demographic characteristics are mainly about the user’s gender (male/female) and age group (children/youth/middle age/old age) and the mark diagrams for record are shown in Table 2. The activity characteristics are about the activity types the user engaged in. Based on the pre-investigation of the case parks and physical activity levels, the common observed recreational activity types are roughly divided into sedentary and moderate–vigorous physical activity (MVPA), containing 12 sub-categories as shown in Table 3. These activities are coded respectively by r1 (sightseeing, sit and stand included), r2 (play cards and Chinese chess), r3 (photography), r4 (fishing), r5 (painting and calligraphy), r6 (sing and play musical instruments), r7 (play for accompanying kids), r8 (fitness), r9 (martial arts), r10 (dancing), r11 (ball sports) and r12 (kick shuttlecock).

Since Guangzhou has a typical monsoon ocean climate in the southern subtropics with a humid, rainy and long summer, the systematic observations of four parks were conducted within sunny and comfortable days during the early autumn in October 2018. This study gathered data on weekdays to compare the RS function performance among the four case parks. After on-site observation, the recorded data were input into Microsoft Excel 2016 for counting the various types of recreational activities and their total user number.

#### 2.3.2. Questionnaire Survey

The questionnaire contained three types of questions: (1) the basic characteristics of recreational behaviour regarding the time and frequency of going to the park, traffic and time taken and length of stay; (2) according to the kind of activity most frequently carried out in the park, score satisfaction on the specific activity experience by the Delphi scale method; and (3) the demographic characteristics, including gender, age range, occupation, marital status and residence.

The study adopted a quota random sampling method. According to the N type amounts of recreational activities in the parks, it collected more than 30 samples for each type of group and less than 30 samples for the small activity group under special circumstances. After the pre-survey, to ensure the quality of the questionnaire collection, one-to-one interviewers assisted the respondents in completing the questionnaire, so the effective rate of the questionnaire was as high as 98.3%. Invalid questionnaires were mainly because some respondents randomly checked answers for the purpose of obtaining gifts and a small number of respondents interrupted the questionnaire midway. In the end, this study obtained 1213 valid questionnaires, including 372, 278, 301 and 262, respectively, from Liuhuahu Park, Renmin Park, Tianhe Park and Zhujiang Park. After cleaning the survey data, IBM SPSS Statistics 22.0 was used for mean analysis to obtain the satisfaction level of various recreational groups.

#### 2.3.3. Recreational Area Calculation

The master plans of parks issued by the managers were updated based on site investigation. Then, we calculated the total land area designed for recreation via the tool of Auto CAD 2016 (Autodesk, Mill Valley, CA, USA).

## 3. Results

### 3.1. Number of Participants (Nri)

**Figure 4:**
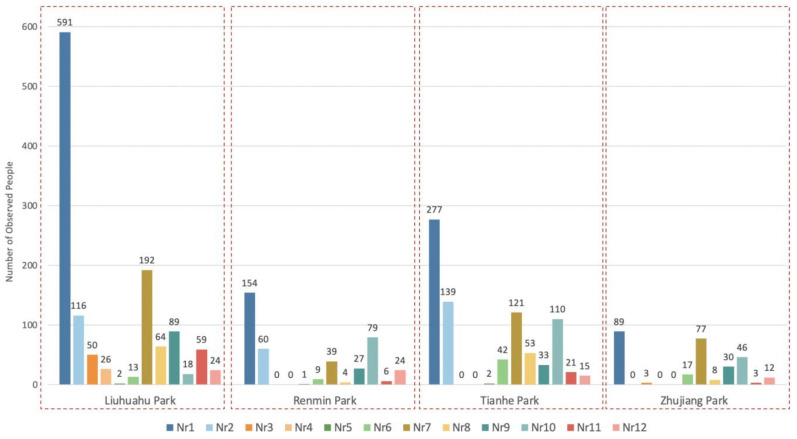
Number of observed people in various activity groups per hour (Nri) in the four parks.

According to the statistical results of the systematic observation data shown in Figure 4, the types of recreational activity carried out in the case parks were similar. Specifically, Liuhuahu Park had the most types of recreational activity, with x = 12 types, followed by Tianhe Park and Renmin Park, with x = 10 types (both lacking r3 photography and r4 fishing) and Zhujiang Park had the least types of recreational activity, with x = 9 types (lacking r4 fishing, r6 play cards and Chinese chess and r7 painting and calligraphy). Furthermore, there were certain differences in the participant amounts of each activity group (*Nri*). The ratios of observed people in various recreational groups in each park were roughly the same. In addition, the spatial distribution of various activity groups and the average total number of users per hour in the case parks are shown in Appendix C.

### 3.2. Average Satisfaction Level ($S_{ri}$ and $\bar{S}$)

According to the satisfaction scores of various recreational activity groups of the case parks (see Appendix D), the average satisfaction level of each group and overall groups in each park were calculated as shown in Table 4. When 0.93 < Sri ≤ 1 (1 for very satisfied), it means most participants are very satisfied; when 0.73 < Sri ≤ 0.93, it means most participants are satisfied; when 0.66 < Sri ≤ 0.73, it means most participants are moderately satisfied; when 0.46 < Sri ≤ 0.66, it means most participants are unsatisfied; when 0.20 < Sri ≤ 0.46, it means most participants are very unsatisfied. It can be seen that the dance group had the highest satisfaction (these groups’ satisfaction levels in the four parks were all above 0.8). The satisfaction levels of the painting and calligraphy group and the kick shuttlecock group were all around or below 0.73. Moreover, the fitness group had the largest difference in satisfaction (the satisfaction levels of fitness groups in Liuhuahu Park and Zhujiang Park were higher, the level in Tianhe Park was 0.73 and in Renmin Park it was lower than 0.6).

The order of the average satisfaction level of overall groups was Zhujiang Park > Liuhuahu Park > Renmin Park > Tianhe Park, with values of 0.85, 0.81, 0.77 and 0.75, respectively. As a result, the activity participants were viewed as satisfied in the four parks. Furthermore, we conducted variance analysis on the average satisfaction level of each activity group from the four parks and the result was *p* = 0.01 (*p* < 0.05), indicating that different parks have an impact on satisfaction.

### 3.3. Performance of Recreational Services (P_R_)

Through the calculation of these park plans, the order of approximate recreational site area (St) was Tianhe Park (36.30 hm^2^) > Zhujiang Park (27.60 hm^2^) > Liuhuahu Park (26.20 hm^2^) > Renmin Park (6.24 hm^2^). The *Sa* of Liuhuahu Park and Renmin Park were valued at 30 m^2^ and that of Tianhe Park and Zhujiang Park valued at 60 m^2^ due to their mountainous landscape. Finally, bringing the above statistical data into the formula of P_R_, the order of the comprehensive RS performance of each park was Renmin Park > Liuhuahu Park > Tianhe Park > Zhujiang Park and the specific values were 0.15, 0.12, 0.10 and 0.06, respectively. As a result, the performance of Renmin Park was the highest and Zhujiang Park was the lowest. However, since all the parks were far from reaching the maximum performance of the designed RS (*P_R_* ≥ 1), it is worthy of consideration by urban managers to further improve their RS efficacy.

## 4. Discussion

### 4.1. Difference of Indicators in Revealing Parks’ Recreational Services

The indicators proposed in the study can evaluate the performance of RS in a park from different perspectives and their evaluation effectiveness is also different. The indicators of $N_{ri}$ and $\bar{S}$ have the same function with traditional measurement methods in revealing recreational opportunities and satisfaction that a park provided. In the case study, the $N_{ri}$ reveals that the recreational activity types in Liuhuahu Park are most rich and in Renmin Park are relatively less. The $\sum N_{ri}$ reveals that Liuhuahu Park attracts more observed users per hour in a day than other parks and Zhujiang Park has the fewest users. The $\bar{S}$ reveals that Zhujiang Park has the highest satisfaction and Tianhe Park the lowest. Based on these indicators, it seems that Liuhuahu Park has the best performance of RS, which provides most recreational opportunities and has good satisfaction. However, the evaluation result of the $P_{R}$ indicator is completely different from the above indicators, which demonstrates that Renmin Park has the highest efficacy of RS. As a whole, the case study has proved the difference of these indicators and the advantage of $P_{R}$ in comparing the comprehensive efficacy of RS in parks.

### 4.2. Inference Based on the Performance Comparison of Parks’ Recreational Services

By comparing the performance measurement results of case parks, some knowledge about the parks’ RS is deduced. According to the results of $N_{ri}$, it is possible to compare if the intended recreational activities are met. The results show the expected recreational activities and some unintended activities, including kicking shuttlecock and painting and calligraphy, are provided in each park. In addition, according to the results of observed people of different activity groups and their graphic spatial distribution (see Figure 4 and Appendix C), the overall recreational spaces of four parks are more applicable for sightseeing and some activities that do not depend on facilities. Together with other studies [56,57,58], the results infer that places with sports courts/facilities for ball sports activities are generally scarce in Chinese parks [48], which results in fewer sports activity groups in parks.

According to the results of $\bar{S}$, Zhujiang Park with beautiful gardens and high-quality management has better satisfaction. It reaffirmed others’ observations [45,59] that ornamental vegetation of the parks can significantly enhance the recreational satisfaction levels of users. Meanwhile, the overall satisfaction levels of the four parks are all above the “satisfied” level, indicating that residents who are willing to use the parks are generally satisfied with the park construction.

According to the results of $P_{R}$, we find that although Renmin Park is not outstanding in relation to user number and satisfaction, its service efficacy is the highest. Previous studies demonstrated that the size of green space has a positive correlation with the user willingness and frequency [30,60,61]. When it comes to the total number of users, large-scale parks with larger recreational spaces and richer activity facilities, such as Liuhuahu Park and Tianhe Park, generally had obvious advantages over smaller ones [6,62]. However, through our quantitative performance comparison, exceptionally Renmin Park as the smallest one had more recreationists than Zhujiang Park with larger recreation areas and it provided RS more efficaciously than other parks. Consistent with the research finding of Kaczynski et al. [63], our study suggests that the area size is not a decisive factor affecting the overall performance of RS in a park, at least for large parks.

### 4.3. The Implication for Refined Park Design and Management

Chinese society has undergone rapid urbanisation. In China, the national population has exceeded 1.4 billion and the urbanisation rate exceeded 60% by 2019 [64]. There are 250 big cities with a population of more than 1 million, among them 20 cities with a population of more than 4 million. With the rapid urban sprawl, the densely settled Chinese cities contribute to the decreased number of outdoor places available for physical activity [65,66]. Faced with the pressure of resource shortage, it is necessary to implement refined design and management of limited urban green spaces and improve the RS efficacy of green space. On the basis of the advantages of large parks in providing RS clarified by previous studies, our findings further provided value implications for the refined design and management of large parks.

First, an important inspiration is that the overall spatial design and configuration have a significant impact on the occurrence and development of various physical activities. We believe that the high RS performance of Renmin Park has something to do with its recreational space system. In a word, although a large park (>5 ha) within a reasonable distance is bound to be more attractive [30], how the park can exert its greatest RS efficacy depends on its internal recreational space system to promote the self-reinforcement process.

Second, the managers should pay more attention to the key performance indicator on the parks’ RS efficacy and not be blinded by the appearance that large parks can attract more users. For example, some studies reported a large portion of the population experienced a deficit of urban green spaces in Guangzhou [14]. However, contradictorily, our study showed a big difference in the actual performance of built parks. That indicates that the mere presence or area size of parks cannot determine that the park is used efficiently [25]. Thus, in addition to the green space quantity, focusing on the actual RS efficacy of built parks is a much more substantive action to benefit public life, especially in the context of land shortage.

### 4.4. Limitations and Future Developments

The assessment indicators this study developed proved to be an effective approach to finely compare the RS differences of urban parks. Though carefully executed, some limitations and future developments of this study deserve further mention.

First, more case studies with efficient investigation methods are needed to strengthen the reliability of the research findings. To obtain deep data on the specific usage of various activities in a park, field observation research is essential. However, due to time, labour and cost constraints of direct field observation and the survey method, this study included a limited number of case parks. Future studies will continue with more case studies and improve the efficiency of investigation by combining unmanned aerial vehicles [67] and online surveys (e.g., Internet-based PPGIS) [6]. Then, we can further carry out scientific statistical analysis on the basis of a large number of samples.

Second, the impact of external factors on the RS of a park is not discussed. It is well known that the land use, population and accessibility around the park also have an important impact on the use of a park. As this study mainly focuses on the functional performance of the park’s internal spaces, in the case selection we chose the adjacent comprehensive parks, which are all within high-density residential areas of the city centre and close to the subway stations, so as to reduce the external differences between the case parks. Nevertheless, the study is still unable to completely avoid the interferences that may exist from external factors. Future studies could further integrate external and internal factors and examine their influence weight and mechanism on the RS function performance of the parks.

## 5. Conclusions

A high-quality urban park has an important influence on residents’ daily recreational behaviour and public health. Compared with conventional evaluation studies on the RS of urban parks, this study proposes a collective set of performance indicators considering the RS function for various types of activities and service efficacy of a park, which can be used for comparing the parks’ differences. With a case study of four urban parks with different spatial attributes in central Guangzhou, China, the performance comparison results showed that (1) the number and satisfaction of recreational visitors cannot reflect the overall RS efficacy of a park; (2) large parks with more facilities can attract more types of physical activities; (3) the larger size of a park can accommodate more people and ornamental vegetation can enhance users’ recreational satisfaction, but the recreational space system affects the efficacy of RS.

With respect to the research method, these indicators can also be implemented in the landscape performance evaluation tool to measure the RS of other urban green spaces. In theory, the performance comparison between different parks can reveal in depth what internal spatial configuration is more effective for RS based on a large number of samples. In the future, performance comparison studies combining a statistical method with large samples of parks are needed to reveal more comprehensively the impact of factors of spatial attributes on the RS, with the intention of providing valuable implications for urban planners, landscape architects and policy makers in refined construction and the renewal of urban parks.

## Figures and Tables

**Table 1:** The amount of park construction of Guangzhou in the main years.

Item	1949	1957	1962	1979	1989	1999	2001	2005	2010	2015	2016	2017	2018	2019
Number of parks (unit)	4	9	19	18	23	68	125	191	232	246	247	247	247	247
Area of parks (hm^2^)	25	168	629	625	959	1824	2797	3230	4562	5193	5198	5198	5198	5198

Note: I. The coverage in this table includes 11 districts. II. The data in this table are from Guangzhou Statistical Yearbook [55].

**Table 2:** The mark diagrams of user demographic characteristics.

Gender	Age			
	Youth	Middle Age	Old Age	Children
Male	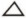	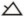	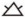	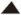
Female	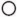	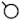	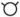	

**Table 3:** The categories, codes and mark diagrams of recreational activities.

Activity Categories	Activity Sub-Categories	Codes	Mark Diagrams
Sedentary	Sightseeing	r1	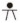
	Play cards and Chinese chess	r2	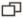
	Photography	r3	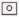
	Fishing	r4	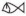
	Painting and calligraphy	r5	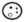
	Sing and play musical instruments	r6	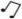
MVPA	Play for accompanying kids	r7	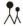
	Fitness	r8	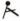
	Martial arts	r9	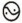
	Dancing	r10	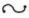
	Ball sports	r11	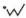
	Kick shuttlecock	r12	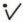

**Table 4:** Average satisfaction levels of each and overall activity groups of the parks.

Parks	Average Satisfaction Levels of Each Activity Group $\left(S_{ri}\right)$												Average Satisfaction Levels of Overall Groups $\left(\bar{S}\right)$
	Sr1	Sr2	Sr3	Sr4	Sr5	Sr6	Sr7	Sr8	Sr9	Sr10	Sr11	Sr12	
Liuhuahu Park	0.86	0.76	0.83	0.81	0.72	0.85	0.72	0.89	0.85	0.83	0.89	0.76	0.81
Renmin Park	0.82	0.74	—	—	—	0.86	0.74	0.57	0.83	0.85	0.76	0.72	0.77
Tianhe Park	0.78	0.8	—	—	0.6	0.78	0.74	0.73	0.77	0.81	0.73	0.72	0.75
Zhujiang Park	0.91	—	0.86	—	—	0.87	0.89	0.8	0.85	0.9	0.78	0.75	0.85

**Figure A5:**
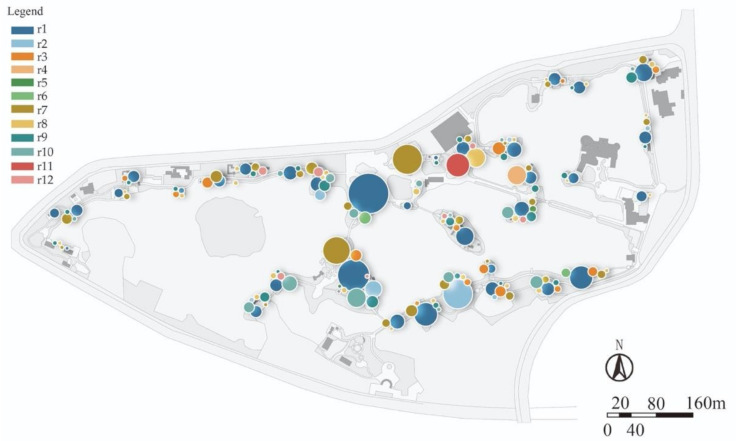
Spatial distribution of various recreational activity users in Liuhuahu Park.

**Figure A6:**
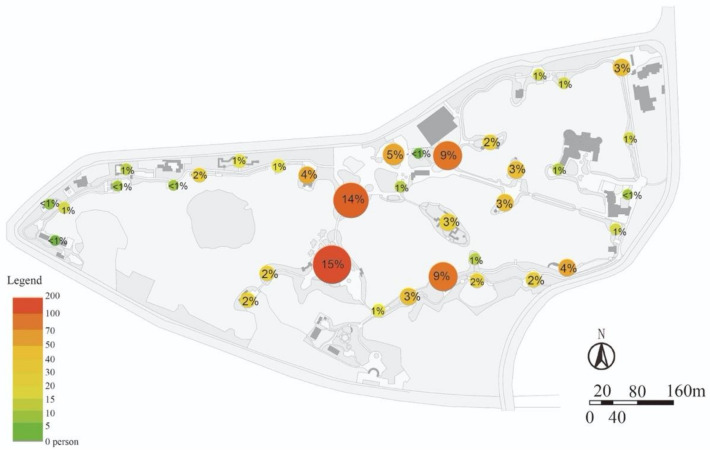
Spatial distribution of average total number of users per hour in Liuhuahu Park.

**Figure A7:**
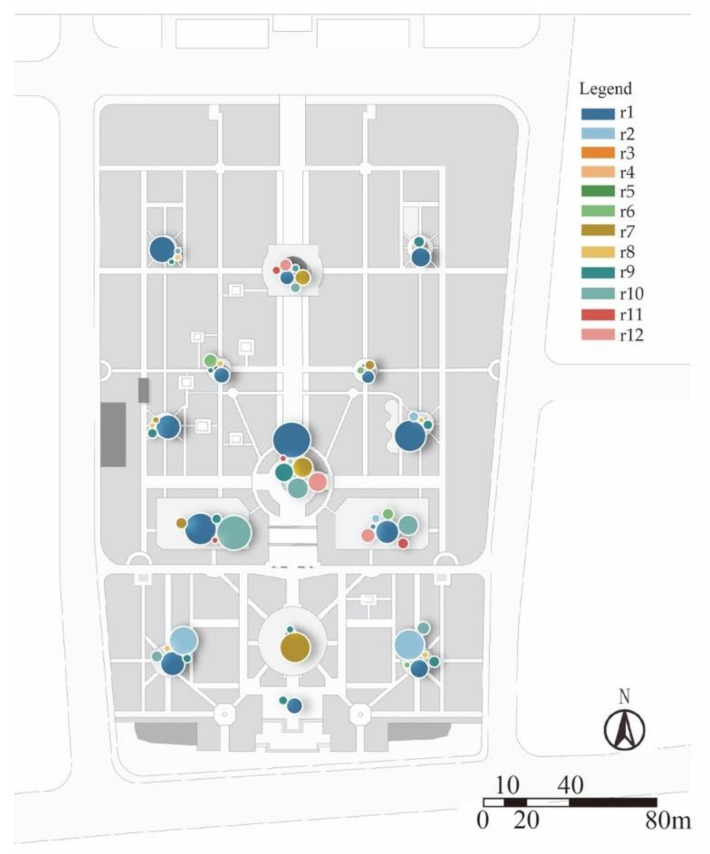
Spatial distribution of various recreational activity users in Renmin Park.

**Figure A8:**
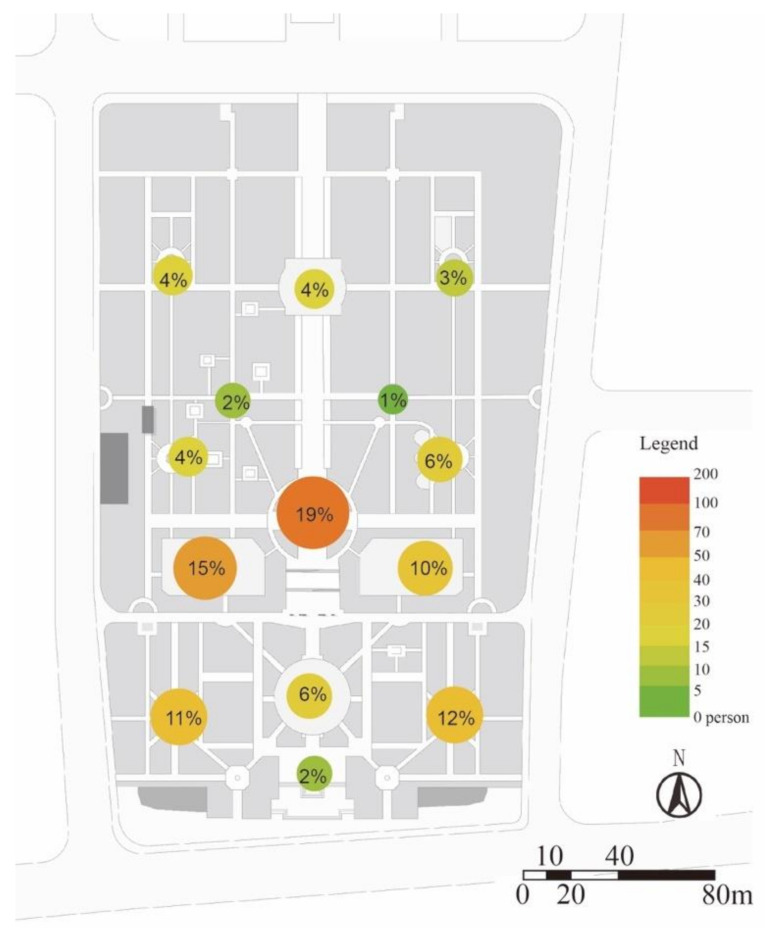
Spatial distribution of average total number of users per hour in Renmin Park.

**Figure A9:**
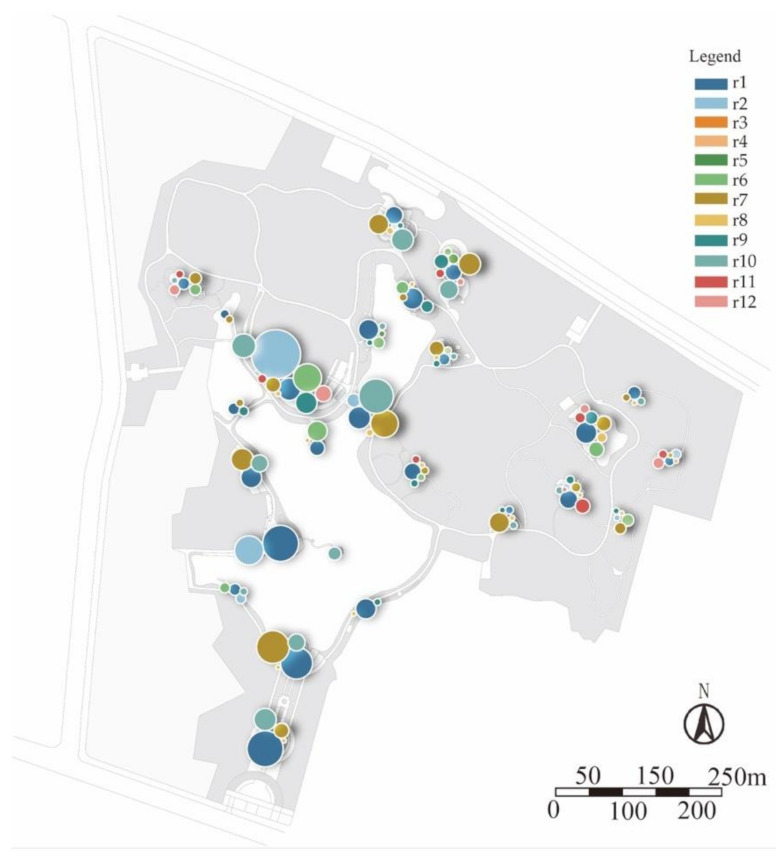
Spatial distribution of various recreational activity users in Tianhe Park.

**Figure A10:**
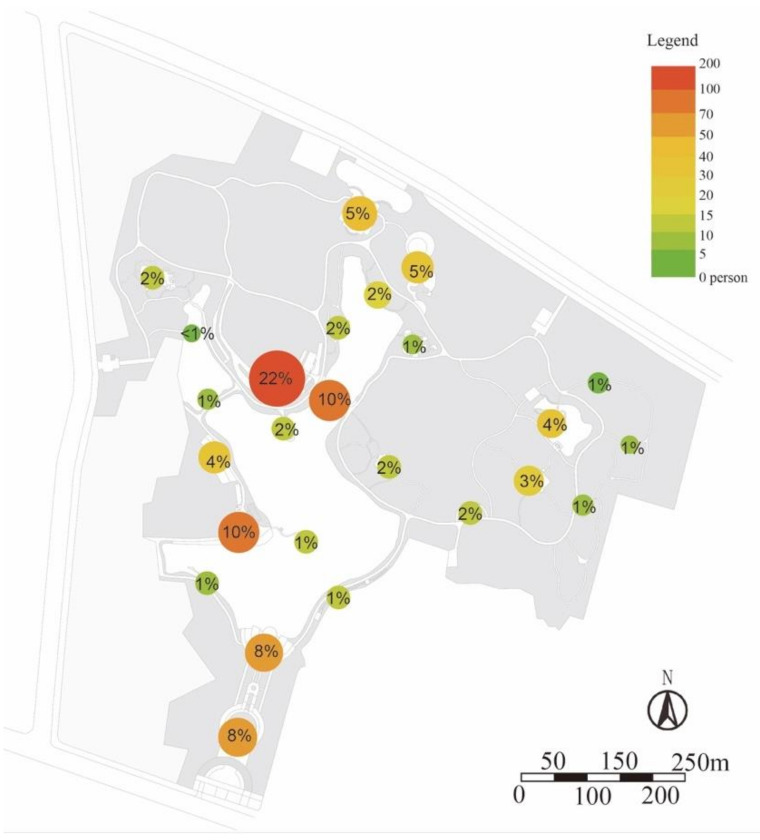
Spatial distribution of average total number of users per hour in Tianhe Park.

**Figure A11:**
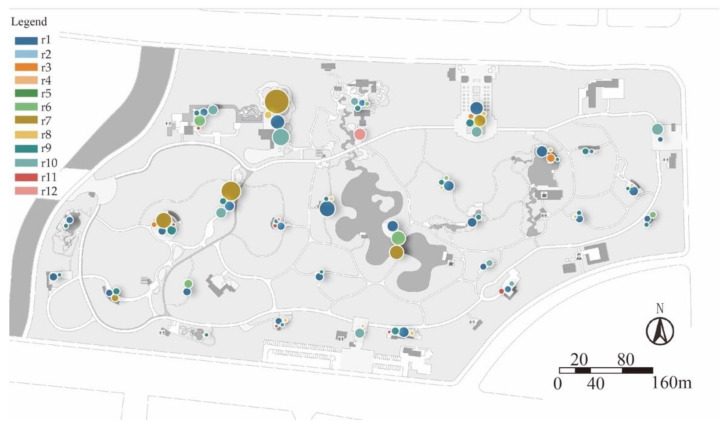
Spatial distribution of various recreational activity users in Zhujiang Park.

**Figure A12:**
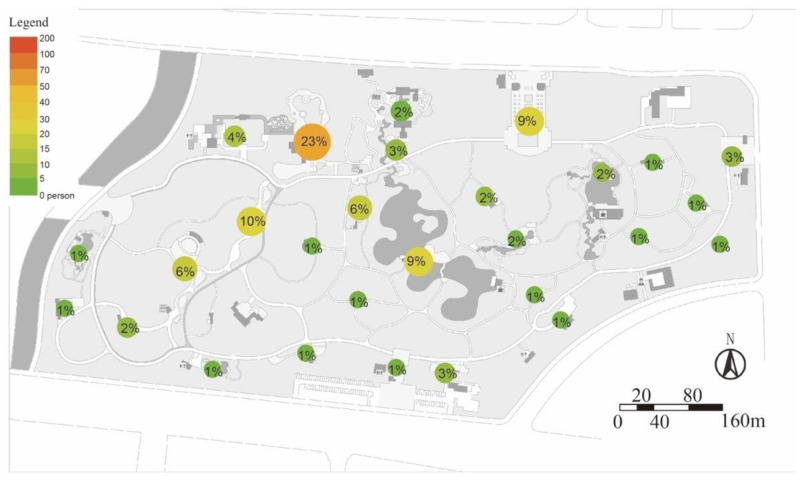
Spatial distribution of average total number of users per hour in Zhujiang Park.

**Figure A13:**
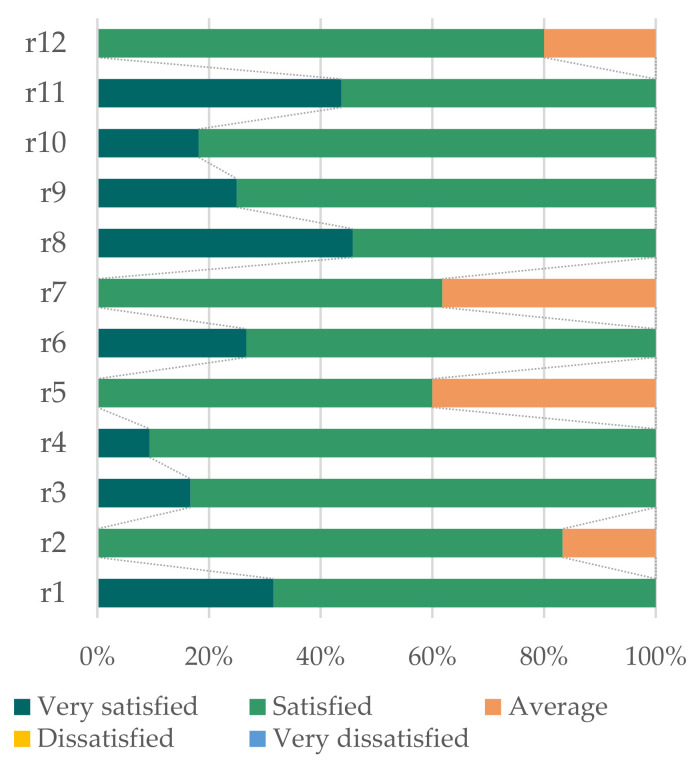
User satisfaction in Liuhuahu Park.

**Figure A14:**
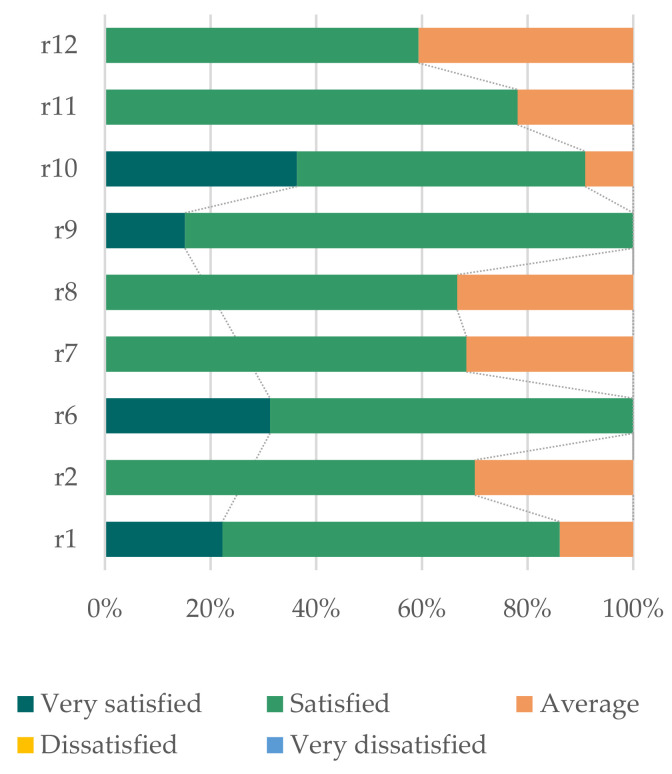
User satisfaction in Renmin Park.

**Figure A15:**
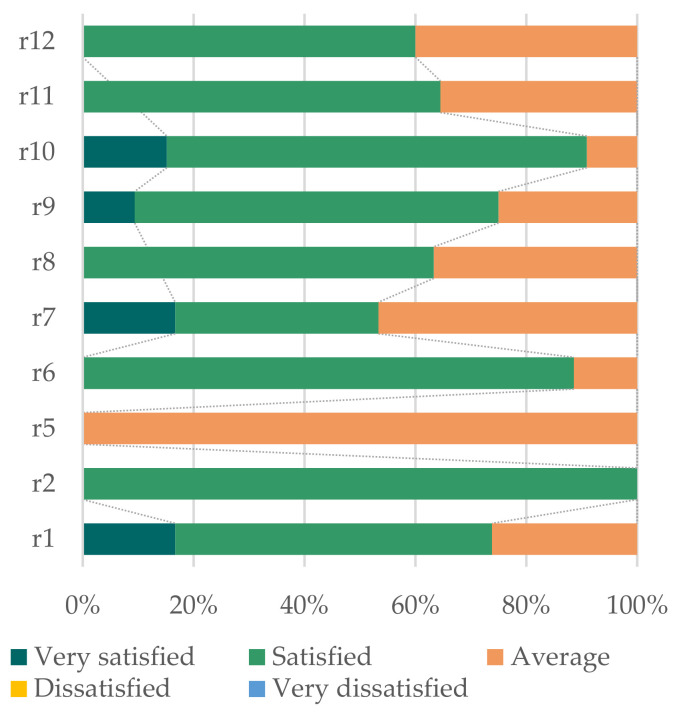
User satisfaction in Tianhe Park.

**Figure A16:**
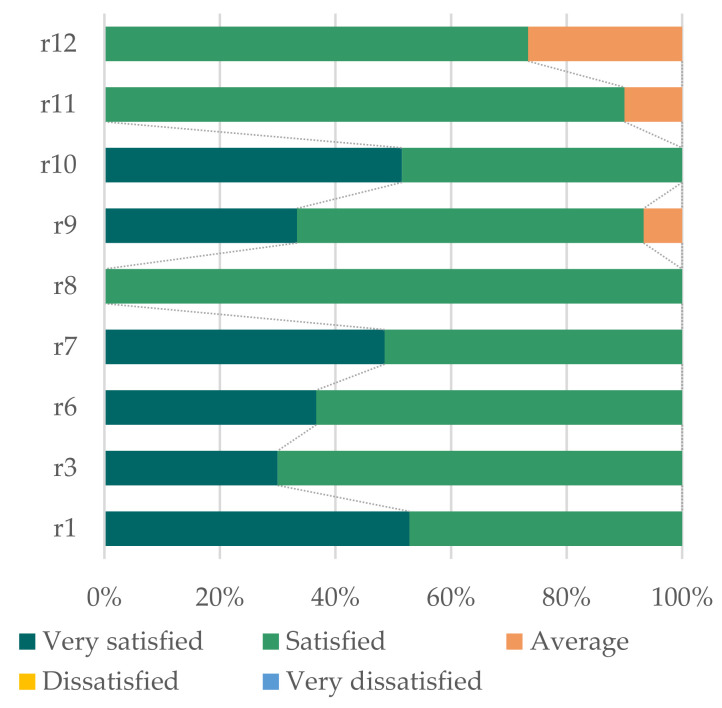
User satisfaction in Zhujiang Park.

## Data Availability

The data presented in this study are available on request from the corresponding author. The data are not publicly available because other studies related to the project are ongoing.

## Appendix B

**Table A1:** Urban park recreation usage observation record sheet template.

XX Park Recreation Usage Observation Record Sheet		
**Recreation space unit codes:**		
** A.** XXX zone **B.** XXX zone **C.** XXX zone ⋯⋯ **N.** XXX zone		
**Recreation space node codes:**		
**a.** landscape architecture		
**b.** square or open space		
**c.** water platform		
**d.** landscape bridge		
**e.** stone setting		
**f.** flower ponds or flower paths		
**g.** landscape paths		
**h.** fitness trails		
**i.** artificial facilities		
**DATE:**		
**PLACE:**		
**WEATHER:**		
**Time range:**		
**Space unit zone**	**Space node**	**User numbers with demographic characteristics and recreational activity engaged in**
A1 (Attached with space nodes of the unit plan)	A1 a1	
⋯	⋯	
NX (Attached with space nodes of the unit plan)	NX nx	
